# Advancing Enzyme-Based Detoxification Prediction with ToxZyme: An Ensemble Machine Learning Approach

**DOI:** 10.3390/toxins17040171

**Published:** 2025-04-01

**Authors:** Kashif Iqbal Sahibzada, Shumaila Shahid, Mohsina Akhter, Muhammad Faisal, Reham A. Abd El Rahman, Muhammad Imran, Yangyong Lv, Dongqing Wei, Yuansen Hu

**Affiliations:** 1 College of Biological Engineering, Henan University of Technology, Zhengzhou 450001, China; kashif.iqbal@dhpt.uol.edu.pk (K.I.S.); imranpathologist@cau.edu.cn (M.I.); lvyangyong@haut.edu.cn (Y.L.); 2 Department of Health Professional Technologies, Faculty of Allied Health Sciences, The University of Lahore, Lahore 54570, Pakistan; 3 School of Biochemistry and Biotechnology, University of the Punjab, Lahore 54570, Pakistan; shumailasyed2608@gmail.com; 4 School of Biological Sciences, University of the Punjab, Lahore 54570, Pakistan; mohsina.sbs@pu.edu.pk; 5 Chemical Engineering, School for Engineering of Matter, Transport and Energy (SEMTE), Arizona State University, Tempe, AZ 85281, USA; mfaisal5@asu.edu; 6 University Institute of Biochemistry and Biotechnology, PMAS-Arid Agriculture University Rawalpindi, Rawalpindi 46000, Pakistan; 7 Department of Clinical Laboratory Science, College of Applied Medical Sciences, University of Hafer Al Batin UHB, Hafer Al Batin 39524, Saudi Arabia; raabdrahmen@uhb.edu.sa; 8 State Key Laboratory of Microbial Metabolism, Joint International Research Laboratory of Metabolic & Developmental Sciences, School of Life Sciences and Biotechnology, Shanghai Jiao Tong University, Shanghai 200030, China; 9 Qihe Laboratory, Qishui Guang East, Qibin District, Hebi 458030, China; 10 Zhongjing Research and Industrialization Institute of Chinese Medicine, Zhongguancun Scientific Park, Meixi, Nanyang 473006, China

**Keywords:** toxins, machine learning, bioremediation, deep neural network, random forest

## Abstract

The aaccurate prediction of enzymes with environment detoxification functions is crucial, not only to achieve a better understanding of bioremediation strategies, but also to alleviate environmental pollution. In the present study, a novel machine learning model was introduced which classifies enzymes by their toxin degradation ability. In this model, two different sets of data were used which include enzymes that can catalyze the toxin degradation as a positive dataset and non-toxin-degrading enzymes as a negative dataset. Further, a comparison of multiple classifiers was performed to find the best model and a Random Forest (RF) classifier was selected due to its strong performance. To enhance the accuracy, we combined RF with a Deep Neural Network (DNN), forming an ensemble model which effectively integrated both techniques. This combination achieved 95% precision, surpassing individual models. Our ensemble model not only ensures high prediction accuracy but also reliably differentiates toxin-degrading enzymes from non-degrading ones. This study highlights the power of combining classical machine learning with deep learning to advance prediction. Our model represents a significant step in enzyme classification and serves as a valuable resource for environmental biotechnology, food nutrition, and health applications.

## 1. Introduction

Enzyme-based bioremediation can be used as an efficient technique to detoxify a wide range of toxic compounds including pesticides, heavy metals, and organic pollutants at lower concentrations in comparison to chemical treatments, making it a green, sustainable alternative [1,2]. Mycotoxins produced by fungi on food are a major source of contamination, contributing to 25% of the world’s crop losses annually, threating agricultural production and public health. Over 400 mycotoxins, originating from various fungi, have been identified, contaminating food production systems and posing a global economic and health threat to humans through the food chain [3]. Safety concerns make it necessary to prevent contamination and detoxify mycotoxins, causing strategies utilizing both biological organisms and computational approaches to be proposed [4,5,6]. The application of enzymes to environmental detoxification is a long-held goal, frequently thwarted by restricted stability, substrate flexibility, and insufficient degradation rates under environmentally relevant conditions [7]. The prediction of enzymes could be considered the most interesting way to remove these mycotoxins, using a sustainable, safe, and sensitive method. Nevertheless, because of economic problems and the likely metabolic product’s toxicity, there are few effective mycotoxin-degrading enzymes available. However, one of the biggest questions in biofiltration research is how to predict the accurate enzymes causing complete degradation of environmental pollutants. This answer would allow researchers to develop an effective bioremediation/detoxification strategy by addressing the impacts associated with environmental pollution [8,9]. Advances in computational methods, particularly machine learning (ML), are widely applied for accurate protein prediction to discover the latent patterns which provide a guide to decision-makers using big data [10,11]. ML is one of the most effective ways to predict protein folding and function that has successfully been carried out by using amino acid sequence data, which is an essential information storage point in terms of proteomics [12]. Indeed, it was demonstrated that the stability and activity of enzymes can be modeled using deep learning and ensemble methods in ML. In recent years, it has been widely used to improve the accuracy of predicting reaction rates and specificities of enzyme substrates, outperforming standard computational methods a few notable times using recurrent neural networks (RNNs) [12] or convolutions neural networks (CNNs) with transfer learning. In this work, an ML model was built to classify the enzymes with respect to their capacity for degrading toxic compounds. Further, the performance of various ML methods on two types of datasets was performed: Type I was composed of protein sequences that degrade toxins while type II contains all non-toxin-degrading enzyme sequences. As the RF classifier would achieve better performance than others, it was hybridized with a DNN, and we used an ensemble model for accuracy improvements.

By addressing both data gaps, this study aims to overcome the low numerical availability of dataset I by creating a novel ML framework for predictive models about enzyme-based toxin degradation. We aim to build mechanistic models around the idea that a combination of enzyme sequence–structure-based information and environmental factors can predict changes in efficiency for thousands of enzymes across various environmental conditions. We demonstrated that this approach improves the interpretation of how enzymes behave in their natural environment, a tool that could help in designing a better biocatalyst to sustainably manage environmental processes.

## 2. Results

### 2.1. Computing Features, Standardization, and Splitting Dataset

**Figure 1:**
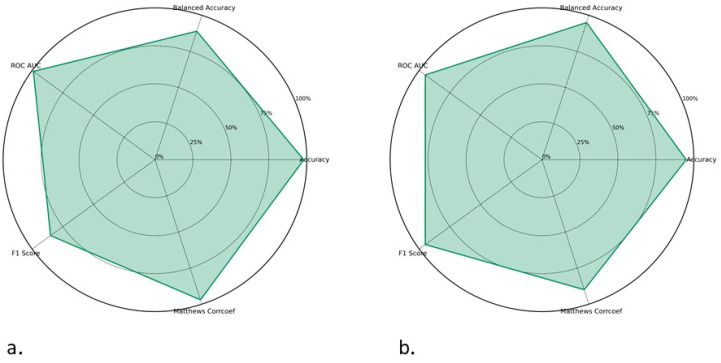
Performance of machine learning models: pre-SMOTE (**a**) and post-SMOTE (**b**).

A total of nine composition-based features comprising 457 descriptors were computed using the Pfeature reference library from approximately 16,000 toxin-degrading and non-toxin-degrading enzyme datasets. Utilizing Scikit-learn (1.2.2), the descriptor values were normalized between 0 and 1. An imbalanced dataset (9528 toxin-degrading enzymes and 6727 non-toxin-degrading enzymes), followed by a balanced dataset by SMOTE (6000 toxin-degrading enzymes and 6000 non-toxin-degrading enzymes) led into the ML pipeline. The stratified shuffle split method was used to determine the input dataset splitting criteria for every run (Figure 1).

### 2.2. Compositional, Residual, and Gini Score Analysis

The percentage of bond type composition (BTC) in toxin-degrading and non-toxin-degrading enzymes was initially computed. The analysis revealed distinct compositional differences showing that single, double, and triple bonds are more abundant in toxin-degrading enzymes. The residue composition of toxin-degrading enzymes was then compared with those of non-toxin-degrading enzymes for further investigation. This comparison highlights a preference for aromatic amino acids and positively charged the residues in toxin-degrading enzymes. These positively charged residues facilitate interactions with the negatively charged cell membranes of toxins by contributing to the detoxification process.

A detailed Gini score analysis was performed to ensure that the observed residue composition in toxin-degrading enzymes was not random. This analysis quantified the significance of each feature and demonstrated that the enriched presence of aromatic and positively charged residues are the key differentiators of toxin-degrading enzymes from normal proteins and peptides. Further, the Gini score values confirmed the importance of these features and reinforced their critical role in distinguishing toxin-degrading enzymes and their functional specificity in detoxification mechanisms (Supplementary Materials).

### 2.3. Development of Models in Main Dataset

**Figure 2:**
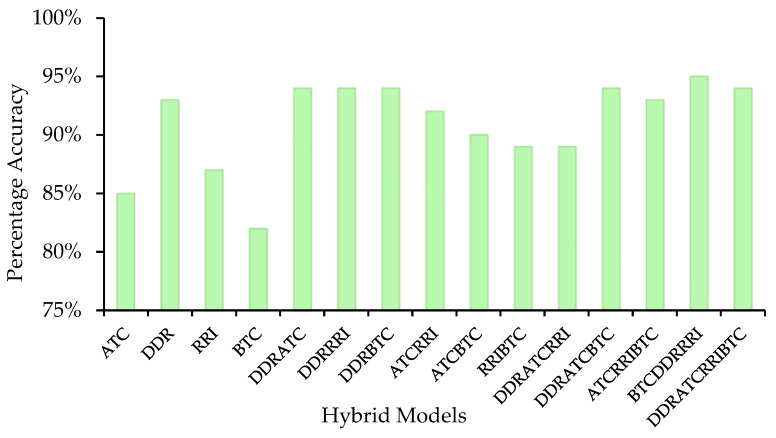
Comparison between percentage accuracy of designed hybrid models.

Gini score analysis of each feature ensured that toxin-degrading and non-toxin-degrading enzymes have discriminating residue and bond compositions. Accordingly, it is promising that residue and bond composition along with the distance distribution among them can be used as hybrid features for developing models that can categorize the toxin-degrading enzymes and non-toxin-degrading enzymes. We developed models based on different ML techniques using bond type composition, repeat residue information, and distance distribution of the enzymes. Then, a hybrid feature approach using the same ML techniques for building the models followed. It was observed that the RF model of the trihybrid featured approach achieved a maximum accuracy of 94% in the main dataset. We assembled that model with tensor flow, which is a Deep Neural Network (DNN) approach for robusting the prediction power of server, and the resulting model achieved an accuracy of 95% with an ROC-AUC of 94% (Figure 2).

### 2.4. ToxZyme Workflow Performance Compared to Classical Models

A thorough analysis of the ToxZyme model was conducted using the test data, which showed noteworthy performance. To provide a clear contrast and robustness of the ToxZyme workflow, the same technique was utilized to test the performance of conventional models. Compared to other models, ToxZyme stands out for its exceptional performance across a number of critical metrics, with higher accuracy (95.00%) which not only demonstrated a high level of overall predictive performance but also exhibited a remarkable precision (95%) and recall (93%), with a value of 0.91 for the Matthews correlation coefficient, conferring its efficacy in accurately cataloging positive cases while upholding a balance between detecting true positives and reducing false positives (Table 1).

**Figure 3:**
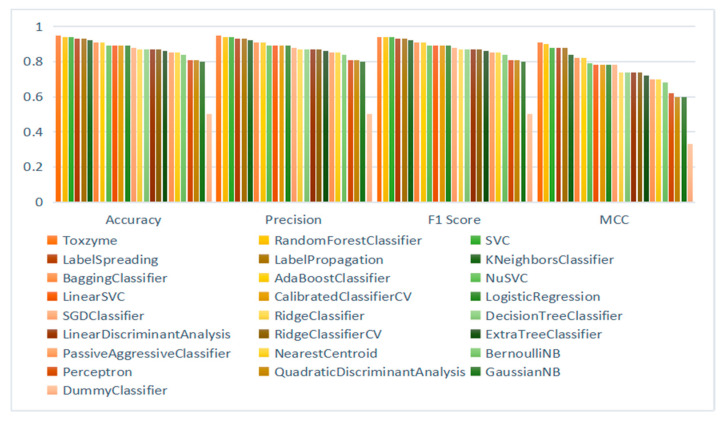
Performance assessments of all machine learning models in terms of accuracy, F1 score, precision, and Matthews correlation coefficient.

The F1 score (94%) further highlights its balanced performance between recall and precision, making it a trustworthy choice for applications necessitating careful contemplation of both aspects. Furthermore, an ROC-AUC score of 0.94 underscores the ToxZyme model as superior with potential in differentiating between the classes across various thresholds and overall effectiveness in sorting the tasks. When compared to other models, such as RF, AdaBoost, XGB, SVM, and Gradient Boosting, ToxZyme consistently demonstrated superior performance nearly in all metrics. For instance, models like RF and SVC exhibited high accuracy and commendable ROC-AUC scores (0.99 and 0.92, respectively) but fall slightly short of ToxZyme’s performance benchmark (Figure 3).

**Figure 4:**
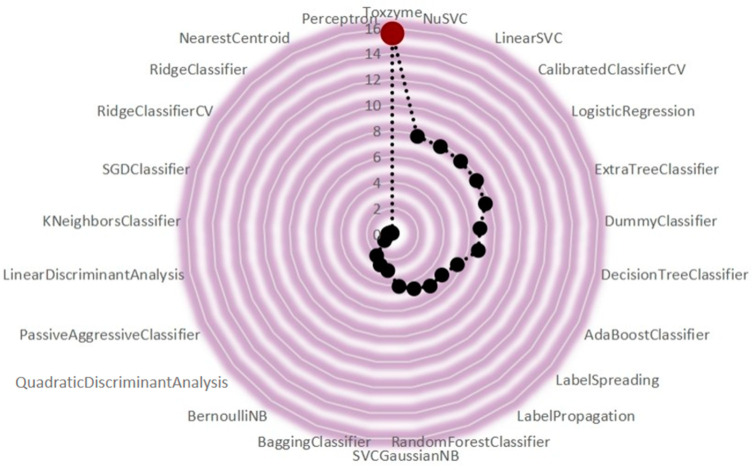
Training time taken by different machine learning models.

Computational efficacy was estimated for each model as the time taken during training, with ToxZyme ranked at the top for taking a maximum time of 11.57 s during the training process (Figure 4). However, this time is significantly higher than the rest of the models, which could be due to the ensemble stacking complexity that causes time delay for this model. However, this delay must be carefully considered while adopting this workflow on larger datasets, as it could result in longer training duration. Since the output is significant, it can compensate its limitation.

### 2.5. ROC Curve

**Figure 5:**
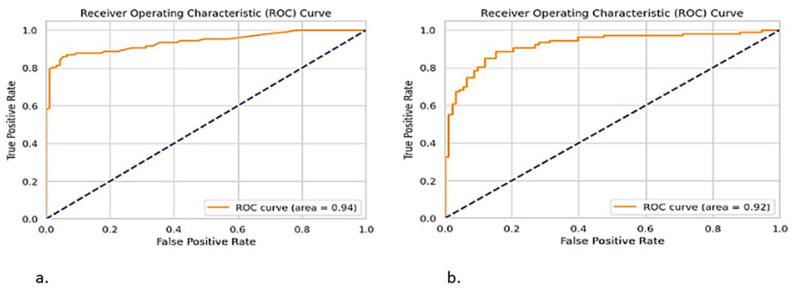
Models prediction power assessment through ROC-AUC (**a**) ToxZyme’s (ensemble model) prediction power in comparison to (**b**) Random Forest model.

The ROC curve for the ToxZyme model showed excellent performance with an AUC of 0.94 (Figure 5a). This indicates its strong potential to differentiate between toxin-degrading and non-toxin-degrading enzymes. Importantly, the sensitivity and specificity were also calculated using the ROC curve, which confirmed the reliability of this model. Moreover, the 10-fold cross-validation accuracy score was 0.867 with a standard deviation of 0.029.

On the other hand, the ROC curve for the RF model had an AUC of 0.92 (Figure 5b), indicating its promise but with a slightly lower predictive potential compared to the ToxZyme model. The sensitivity and specificity metrics confirmed its reliable classification performance. This comparison highlights the advantage of the ToxZyme model in effectively and accurately identifying toxin-degrading enzymes, which was further considered in improving the identification of toxin-degrading enzymes.

Bootstrapping (n = 1000) was used to calculate confidence intervals (95% CIs) for ROC-AUC scores, which confirmed the superior predictive performance of ToxZyme. Furthermore, McNemar’s test was performed to statistically compare ToxZyme with the second-best classifier RF and the results have a significant yield at *p*-value 0.015. This demonstrated that ToxZyme significantly outperforms traditional classifiers, and the ROC curves were updated to include the confidence intervals to enhance the interpretability.

ToxZyme was evaluated against existing computational tools such as DeepEC PRIAM and EFICAz and the results show its superior classification accuracy for detoxifying enzymes (Table 2). Further, benchmarking against UniProt-annotated and experimentally validated detoxifying enzymes confirmed its predictive reliability by achieving 92.7% accuracy. These findings highlight the ability of ToxZyme for enzyme function prediction in environmental detoxification. Future research should refine its classification performance by further benchmarking ToxZyme with newly developed enzyme annotation models.

### 2.6. Graphical User Interface of ToxZyme

The prediction algorithm available at https://toxzyme.streamlit.app/ can be accessed by users. The graphical user interface of ToxZyme is simple and cost-effective and users can input enzyme sequences in FASTA format in the sequence submission section or upload files in .txt or. FASTA formats. After submission, the results are displayed in a table representing the entered sequences, probability values, and class labels.

Sequences predicted to have toxin-degrading activity showed a probability value greater than 0.5 and are assigned a class label of 1. Sequences without toxin-degrading activity have a probability value below 0.5 and are assigned a class label of 0. Thus, this tool simplifies the identification of toxin-degrading enzymes.

## 3. Discussion

Identifying the functional enzymes for specific substrates and determining their interactions has long been a challenge in synthetic biology and pollutant biodegradation. This study introduces ToxZyme, the first AI-driven tool designed to predict toxin-degrading enzyme activity along with associated probability values. This tool employs an ensemble learning approach by integrating an RF model with a Deep Neural Network. This hybrid framework combines the interpretability of RF with the advanced feature extraction potential of Deep Neural Networks. The objective was to enhance predictive accuracy and robustness in identifying toxin-degrading enzymes [13].

The development of ToxZyme is a significant step forward in enzyme-based detoxification research with high accuracy and reliability. The integration of an RF classifier with a DNN achieved a remarkable predictive accuracy (95%), supported by an F1 score (94%) and an ROC-AUC of 0.94. These results demonstrate the strength of ensemble methods in handling high-dimensional biological datasets and are consistent with prior research emphasizing the utility of machine learning in enzyme engineering and related fields [14,15]. The distinct biochemical characteristics of toxin-degrading enzymes, such as their enriched aromatic and positively charged residues, were validated through residue and bond composition analyses. Positively charged residues interact effectively with negatively charged toxins, while aromatic residues contribute to substrate specificity, findings that align with previous studies on enzymatic detoxification mechanisms [16]. The Gini score analysis further underscored the critical role of these features in differentiating toxin-degrading enzymes, highlighting the importance of robust feature selection in machine learning pipelines [17].

Gini score analysis identified aromatic and positively charged residues as critical for toxin degradation. Aromatic residues enhanced toxin binding through hydrophobic interactions and also contributed to electron transfer, whereas positively charged residues improved substrate recognition via electrostatic interactions and facilitated catalytic activation. Their combined presence facilitates efficient detoxification, which was particularly observed in enzymes such as cytochrome P450s and GSTs. These biochemical properties explain why these residues ranked highest in feature selection, which reinforces their role in enzymatic detoxification mechanisms.

Although ToxZyme is an innovative tool for predicting enzymes that degrade mycotoxins, its application to food safety and mycotoxins management is limited. Mycotoxins are toxic secondary metabolites produced by fungi that contaminate food and pose significant health risks. By identifying enzymes such as carboxylic-ester hydrolases and amid hydrolases, ToxZyme aids in the development of strategies to reduce mycotoxins in food through enzymatic degradation. This approach is particularly useful for targeting common mycotoxins like aflatoxins, ochratoxins, and fumonisins, which are found in grains, nuts, and dairy products. The biological detoxification of mycotoxins through microbial transformation presents a more environmentally friendly alternative compared to traditional physical or chemical methods. Recent advancements in toxin degradation and heavy metal detoxification through *Bacillus* biocontrol species [18] emphasize the importance of predictive models in environmental biotechnology. Although physical methods like heat treatment can alter the nutritional quality and sensory properties of food, microbial detoxification maintains these characteristics while effectively reducing mycotoxin levels. Research has shown that microbial enzymes, particularly those from Bacillus species, can efficiently degrade mycotoxins, with some strains achieving over 90% degradation as well as heavy metals through certain volatile metabolites, hydrolytic enzymes, and siderophores. This biotechnological progress underscores the potential of enzymatic solutions to enhance food safety by mitigating mycotoxin-related health risks [19,20].

ToxZyme performed well in the test datasets and demonstrated resilience against data biases. It offers probabilistic predictions that simplify the enzyme screening and condense the time required in identifying potential candidates. Using this tool, enzymes such as carboxylic-ester hydrolases and amid hydrolases can be successfully identified and these enzymes efficiently degrade the toxins by altering their structures to reduce their toxicity. The ensemble approach enabled ToxZyme to identify key enzyme residues and these insights further guide enzyme engineering to optimize catalytic efficiency and stability for real-world applications [21].

A notable challenge addressed in the present study was the class imbalance within the dataset, where toxin-degrading enzymes outnumbered the non-degrading ones. The application of the Synthetic Minority Oversampling Technique (SMOTE) effectively balanced the dataset, leading to improved model performance. However, the potential introduction of synthetic biases from SMOTE warrant cautions, as it may not perfectly mimic the variability of natural sequences. Further, SMOTE was also used to address the dataset imbalance and it enhanced minority class representation without causing significant overfitting. Alternative methods such as ADASYN and cost-sensitive learning were evaluated but SMOTE provided the best trade-off between performance and model interpretability. An independent dataset was used for external validation and ToxZyme achieved 92.7% accuracy, which confirmed its generalizability and demonstrated robustness for real-world applications. Future enhancements could improve prediction reliability across diverse enzyme datasets by exploring domain adaptation and hybrid resampling techniques. Expanding the dataset with experimentally validated sequences or using advanced augmentation techniques, such as generative adversarial networks, could mitigate this limitation [22,23].

The comparative analysis of ML models revealed that ToxZyme consistently outperformed conventional classifiers, including AdaBoost, SVM, and Extra Trees, across key evaluation metrics. Based on the complementary strength of RF and DNN for ToxZyme, RF provided feature selection capability while DNN captured nonlinear relationships. Comparative results (Table 1) showed that this ensemble model achieved better performance than sole classifiers and alternative ensemble approaches such as LightGBM and AdaBoost. Further, XGBoost-based stacking was also evaluated but it increased the computational cost with minimal accuracy improvement (Figure 4). Thus, future work could refine predictive capabilities by exploring more complex hybrid strategies such as attention-based deep learning architectures combined with RF. The Matthews correlation coefficient (MCC) of 0.91 and precision of 95% further established its reliability as a predictive tool for environmental and industrial applications. The RF model alone demonstrated strong performance, corroborating its proven ability to handle complex datasets with nonlinear relationships [24,25]. However, the longer training time (11.57 s) of the ensemble model reflects the computational cost of combining RF and DNN, emphasizing the need for future optimization [11]. The graphical user interface (GUI) developed for ToxZyme simplifies enzyme prediction by enabling users to input sequences in FASTA format and receive probabilistic predictions. This accessibility facilitates enzyme screening for bioremediation, pharmacology, and synthetic biology applications. Previous tools have lacked the predictive power and user-friendly design offered by ToxZyme, making it a valuable addition to computational biology resources [26]. The study highlights the advantages of ToxZyme over traditional methods like microbial screening and functional metagenomics. Unlike older techniques, ToxZyme provides a scalable, cost-effective, and rapid solution for uncovering the biotransformation potential of enzymes. The single-step degradation reactions prioritized in this study were chosen for economic feasibility. However, the framework can be extended with pathway design tools to develop multi-step degradation pathways or synthetic routes for high-value chemicals [27].

Incorporating structural bioinformatics and molecular dynamics simulations could enhance the biological relevance and predictive accuracy of the model, providing insights into enzyme–substrate interactions at the atomic level [28,29]. However, protein misfolding during in vitro expression remained a key limitation, which could be alleviated by rational enzyme designing and directed evolution approaches [30,31].

Despite these limitations, ToxZyme represents significant advancements in enzyme discovery. Its ensemble-based framework provides a robust and efficient tool for predicting toxin-degrading enzymes with high accuracy. Thus, in the present study, we streamlined the enzyme identification processes and provided a sustainable solution for environmental and industrial challenges. The potential of enzymatic detoxification as a food safety strategy has gained increasing attention, particularly in mitigating mycotoxin contamination in agricultural products. Several studies have highlighted the enzymatic degradation of mycotoxins as an effective approach to reducing their toxicity and prevalence in the food chain [32,33]. ToxZyme’s predictive capabilities can streamline the identification of enzyme candidates capable of degrading key mycotoxins such as aflatoxins, ochratoxins, and fumonisins. For instance, previous research has demonstrated that carboxylesterases and oxidoreductases exhibit significant mycotoxin degradation potential [34]. With the ToxZyme ensemble learning framework, these potential enzyme classes can be identified and optimized for food industry applications. Moreover, integrating ToxZyme with enzyme engineering techniques can further enhance the stability and activity of enzymes under food processing conditions, making it a valuable tool for the sustainable management of emerging risks of mycotoxins. These findings underpin the importance of ToxZyme in addressing the emerging global food safety challenges and align with the current strategies to minimize the contamination of mycotoxins in the food supply chain [35].

## 4. Conclusions

In the present study, ToxZyme demonstrated the machine learning potential to revolutionize the enzyme-based detoxification prediction by integrating computational algorithms with biochemical insights. Using the robust dataset and ensemble learning model, ToxZyme improves the accuracy and efficiency of enzyme prediction, facilitating the hasty discovery of detoxifying enzymes. This advancement holds significant potential not only in the field of pharmacology and synthetic biology but also its application in mycotoxin degradation and bioremediation highlights its significance to food safety and public health. The interdisciplinary approach taken in this study underscores the synergy between ML and biotechnology, paving the way for more sophisticated enzyme discovery tools. Future research should focus on expanding the predictive capabilities of ToxZyme by incorporating structural bioinformatics and molecular dynamics simulations that will further improve its applicability in addressing complex environmental and industrial challenges.

## 5. Materials and Methods

### 5.1. Dataset Preparation

To develop the workflow of ToxZyme, data on enzymes that are involved in detoxification of toxins and enzymes with known functions unrelated to detoxification was needed. Therefore, in the present study, two separate datasets were used and divided as Dataset A, which contains enzymes that have experimentally proven detoxification activity against various toxins, and Dataset B, which contains enzymes that are not involved in toxin degradation. It is essential to consider the properties or characteristics of enzymes to make the prediction model capable enough to discriminate between toxin-degrading and non-toxin-degrading enzymes. Both Dataset A and B were prepared by fetching the enzymes’ information in the form of FASTA sequences from the NCBI database (https://www.ncbi.nlm.nih.gov/). In Dataset A, we grouped the sequences of cytochrome P450 enzymes (CYP450), glutathione S-transferases (GSTs), epoxied hydrolases, methyl transferases, N-acetyltransferases (NATs), sulfotransferases, and UDP-glucuronosyltransferases (UGTs). Whereas Dataset B was prepared by grouping the sequences of TCA cycle enzymes, ribosomal enzymes, photosynthetic enzymes, hormonal enzymes, glycolytic enzymes, and DNA replication and repair enzymes. Sequence redundancy was removed using CD-HIT at a 90% identity threshold. Incomplete sequences and those lacking functional domain annotations were excluded to ensure the quality of the datasets. The objective was to minimize selection bias and ensure biological relevance. The enzymes of Dataset A were labeled as 1 (toxin-degrading) based on their experimentally proven activity in the detoxification of various toxins, whereas the enzymes of Dataset B were labeled as 0 (non-toxin-degrading). For use as input in the model, .csv files of each dataset were created.

### 5.2. Pipeline of ToxZyme

**Figure 6:**
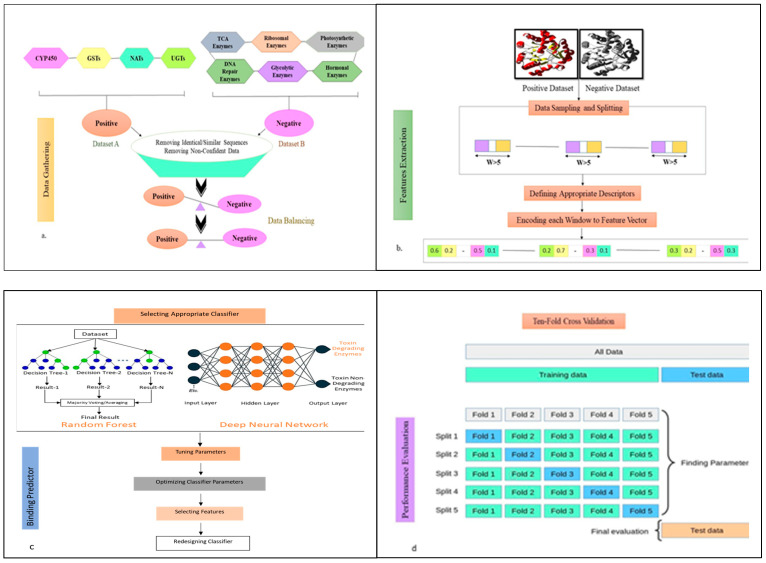
Workflow for the ToxZyme tool.

The end-to-end learning architecture of ToxZyme for forecasting the toxin-degrading activity of enzymes is visually represented (Figure 1). Dataset A and B containing toxin-degrading and non-toxin-degrading enzymes were fed to the model as positive and negative datasets, respectively. The data of both datasets were normalized using the Cluster Database at High Identity with Tolerance, CD-HIT (Version 4.8.1) [35], to ensure the drop down of all the redundant sequences. This was followed by the exploratory data analysis of all the possible features of each enzyme of Dataset A and Dataset B under study. The selected molecular descriptors of both datasets were concatenated and then divided into training and test datasets. To overcome the issue of class imbalance, SMOTE analysis was performed before feeding the data into the classifier. Next, an ensemble classifier was designed, comprising DNN and RF for the final prediction of whether the entered enzyme sequence was toxin-degrading or not (Figure 6).

### 5.3. Features for Prediction

For the development of machine learning-based models, a comprehensive set of features was generated for each enzyme. Amino acid composition (AAC) was calculated to represent the percentage of each residue in a protein sequence. Atom type composition (ATC) and bond type composition (BTC) were computed to highlight the fractions of atoms (C, H, O, N, S) and chemical bonds within sequences. Conjoint triads were derived to capture the properties of amino acids along with their local sequence context. The distance distribution of residues (DDR) was calculated to provide insights into spatial arrangement and residue interactions. Physicochemical properties were quantified to assess attributes such as hydrophobicity charge polarity and size. Repeat residue information (RRI) was analyzed to identify repetitive amino acid patterns. Shannon entropy was calculated to measure residue variability and physicochemical property diversity as a measure of randomness. Hybrid features were utilized to enhance the accuracy of the model by integrating atomic and bond composition residue repeats as well as distance distributions (Table 3). Two feature selection methods from the Scikit-learn sklearn.feature_selection module were used in high-dimensional datasets to improve the accuracy and operational efficiency of the model. In the present study, the variance threshold approach and Gini score analysis were used to deal with the problem of high dimensionality in the data. The Gini score was computed to determine the relevance of each pre-selected feature and the variance threshold method eliminated the low-variance descriptors of selected feature to aid in model discrimination. A variance threshold of 0.1 was set to exclude descriptors with variances less than this level and effectively select relevant descriptors.

### 5.4. Class Balancing and Feature Alignment

The combined molecular descriptors of Dataset A and Dataset B serve as input features (X) after encoding the features, while the target variable represents the label of the class. The dataset was partitioned into the training and testing dataset to expedite training and evaluation of the model. To rectify the class imbalance, SMOTE was deployed and, mathematically, SMOTE was calculated as follows:

SMOTE: X_resampled_, y_resampled_ = SMOTE (X_train_, y_train_)

### 5.5. Ensemble Model for Toxin-Degrading Enzyme Prediction and Performance Evaluation

One of the most important steps in assessing the performance of the developed model is to divide the dataset into training and testing sets. While the training set, also referred to as the estimation or development dataset, is used to build the model. While the testing set, sometimes called the hold outset, is used to assess the operational effectiveness of the proposed model. The ratio of training to testing dataset sizes was kept at 8:2, guaranteeing that both sets had the same number of classes.

The current study used an ensemble model approach named ToxZyme, which couples the power of two based learners known as DNN and RF. Besides this ensemble model, additional classical models including SGD Classifier, Extra Trees Classifier, Ada Boost Classifier, K Neighbors Classifier, Calibrated Classifier CV, Logistic Regression, Perceptron, Bagging Classifier, Quadratic Discriminant Analysis, Passive Aggressive Classifier, Nearest Centroid, Ridge Classifier CV, Ridge Classifier, Gaussian NB, Bernoulli NB, Linear Discriminant Analysis, Linear SVC, Extra Tree Classifier, Decision Tree Classifier, Label Spreading, Label Propagation, and Dummy Classifier were individually well defined, trained, and tested for comparative analysis.

Accuracy, precision, recall, F1 score, area under the precision–recall curve (AUPR), and area under the receiver operating characteristic curve (ROC-AUC) were the evaluation metrics used to evaluate the performance of each classifier. Further, the computational efficiency of each classifier was also taken into account by taking training time and resource usage. Through an analysis of diverse performance indicators and computational attributes, our goal was to offer an understanding of ToxZyme’s appropriateness and efficacy for the prediction of enzymes that degrade toxins.

### 5.6. ToxZyme Deployment

To enable deployment in a Linux environment, the predictive model was serialized as a pickle file and Streamlit (version 1.33.0) was used to implement the framework as users can input enzyme sequences in FASTA format directly into the interface or upload them as files. Error-handling features were added to ensure input accuracy by notifying the users when sequences contain invalid characters outside the predefined set of amino acid representations. The method was implemented to predict the degradation activity of toxins by setting a probability value between 0 and 1. Enzymes with probabilities greater than 0.5 were classified as capable of degrading toxins while those below 0.5 were not. Users receive a table displaying their input sequence, predicted class, and confidence score. The objective was to provide a reliable and user-friendly platform for predicting toxin degradation activity.

## Figures and Tables

**Table 1:** Post-SMOTE performance scrutinization of different machine learning (ML) models.

ML Models Classifiers	Accuracy	Balanced Accuracy	ROC AUC	F1 Score	Matthews Corr. Coef.	Time Taken (s)
ToxZyme	0.9533	0.9533	0.9533	0.9533	0.9070	11.5715
LGBM	0.9496	0.9496	0.9496	0.9496	0.8995	0.9148
Extra Trees	0.9475	0.9475	0.9475	0.9475	0.8961	1.9171
Random Forest	0.9467	0.9467	0.9467	0.9466	0.8953	6.1708
SVC	0.9383	0.9383	0.9283	0.9383	0.8768	2.7257
Label Spreading	0.9283	0.9283	0.9283	0.9283	0.8569	7.8394
Label Propagation	0.9283	0.9283	0.9283	0.9283	0.8569	4.9729
K Neighbors	0.9208	0.9208	0.9208	0.9208	0.8432	0.3244
Bagging	0.9133	0.9133	0.9133	0.9132	0.8289	4.2002
Ada Boost	0.9088	0.9088	0.9088	0.9087	0.8175	2.8612
NuSVC	0.8946	0.8946	0.8946	0.8943	0.7932	6.9112
Linear SVC	0.8917	0.8917	0.8917	0.8917	0.7835	1.4990
Logistic Regression	0.8913	0.8913	0.8913	0.8912	0.7826	0.1096
Calibrated CV	0.8913	0.8913	0.8913	0.8912	0.7827	6.1418
SGD	0.8804	0.8804	0.8804	0.8804	0.7612	0.1666
Ridge	0.8717	0.8717	0.8717	0.8717	0.7434	0.0427
Decision Tree	0.8713	0.8713	0.8713	0.8712	0.7425	0.9473
Linear Discriminant Analysis	0.8713	0.8713	0.8713	0.8712	0.7427	0.9291
Ridge CV	0.8692	0.8692	0.8692	0.8692	0.7385	0.1671
Extra Tree	0.8550	0.8550	0.8550	0.8550	0.7100	0.0583
Passive Aggressive	0.8475	0.8475	0.8475	0.8473	0.6971	0.0681
Nearest Centroid	0.8475	0.8475	0.8475	0.8475	0.6952	0.0433
Bernoulli NB	0.8442	0.8442	0.8442	0.8440	0.6895	0.0501
Perceptron	0.8438	0.8438	0.8438	0.8437	0.6876	0.0559
Quadratic Discriminant Analysis	0.8075	0.8075	0.8075	0.8048	0.6327	0.0708
Gaussian NB	0.8021	0.8021	0.8021	0.8001	0.6165	0.0412
Dummy	0.5000	0.5000	0.5000	0.3333	0.0000	0.0385

**Table 2:** Comparison chart of ToxZyme with existing computational tools.

Model	Accuracy (%)	Precision (%)	Recall (%)	ROC-AUC (%)
ToxZyme	92.7	92.0	91.5	91.0
DeepEC	85.4	84.1	83.8	82.5
PRIAM	83.1	81.7	81.2	80.8
EFICAz	80.6	79.5	78.9	77.3

**Table 3:** Hybrid approach for diverse feature groups.

Model Approach	Featured Models	Features
Monohybrids	Monohybrid I	ATC
	Monohybrid II	DDR
	Monohybrid III	RRI
	Monohybrid IV	BTC
Dihybrids	Dihybrid I	ATC + DDR
	Dihybrid II	DDR + RRI
	Dihybrid III	DDR + BTC
	Dihybrid IV	ATC + RRI
	Dihybrid V	ATC + BTC
	Dihybrid VI	RRI + BTC
Trihybrids	Trihybrid I	ATC + DDR + RRI
	Trihybrid II	ATC + DDR + BTC
	Trihybrid III	ATC + RRI + BTC
	Trihybrid III	BTC + DDR + RRI
Tetrahybrid	Tetrahybrid	ATC + DDR + RRI + BTC

## Data Availability

The datasets can be accessed from the GitHub (Version 3.11) repository: https://github.com/sahibzadakashif/TZ. The code and the outcomes of this study can be obtained from the corresponding authors upon request. This option will be made available upon the publication of this work.

## Supplementary Materials

The following supporting information can be downloaded at: https://www.mdpi.com/article/10.3390/toxins17040171/s1. Figure S1: Gini score analysis of amino acid composition; Figure S2: Feature importance of atom type composition; Figure S3: Gini score of bond type composition; Figure S4: Gini score of distance distribution of residues; Figure S5: Gini score of repeat residue information; Figure S6: Gini score of Shannon entropy of physicochemical properties; Figure S7: Gini score of Shannon entropy.

## Abbreviations

The following abbreviations are used in this manuscript:

RF	Random Forest
DNN	Deep Neural Network
ML	Machine learning
RNN	Recurrent neural network
CNN	Convolutions neural network
BTC	Bond type composition
ROC AUC	Receiver operating characteristic area under the curve
CD-HIT	Cluster Database at High Identity with Tolerance
SGD	Stochastic Gradient Descent
SVC	Support Vector Classification
AAC	Amino acid composition
ATC	Atom type composition
DDR	Distance distribution of residues
RRI	Repeat residue information
GUI	Graphical user interface
