# Supplementary material for: Advancing Enzyme-Based Detoxification Prediction with ToxZyme: An Ensemble Machine Learning Approach
Source: Toxins (Basel). 2025 Apr 1;17(4):171. doi: 10.3390/toxins17040171 (PMC12031443; doi:10.3390/toxins17040171)
Supplement: Supplementary file 1 [file toxins-17-00171-s001.zip › toxins-3475588-supplementary.pdf]

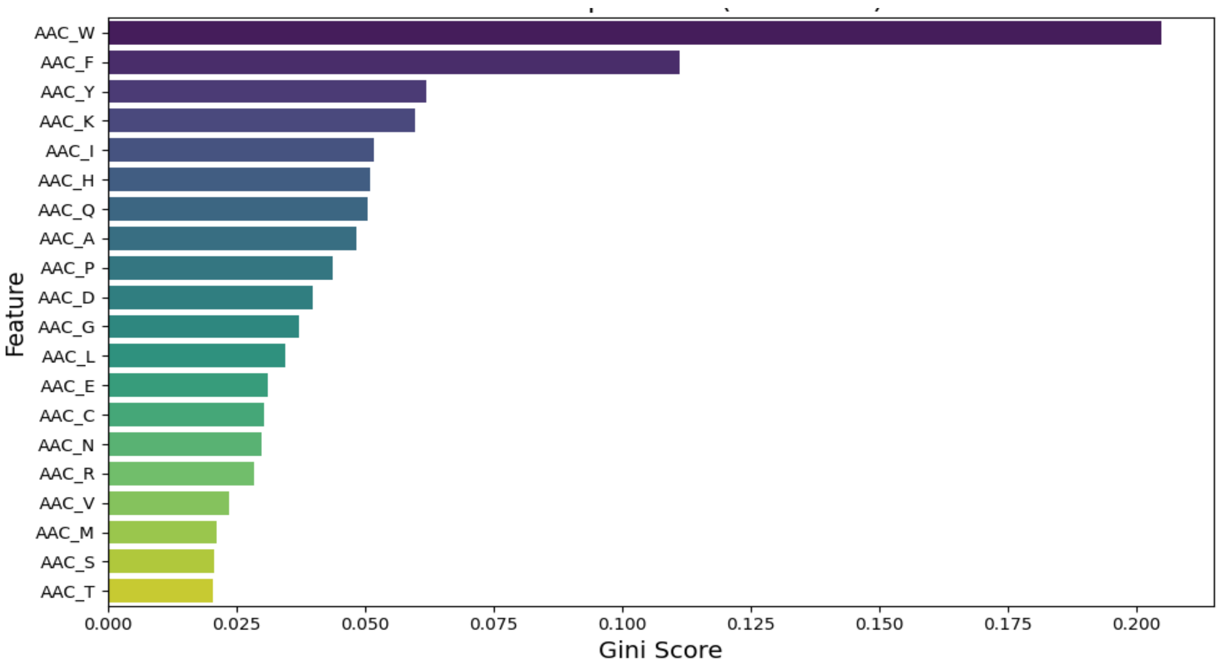

Figure S1: Gini Score Analysis of Amino Acid Composition

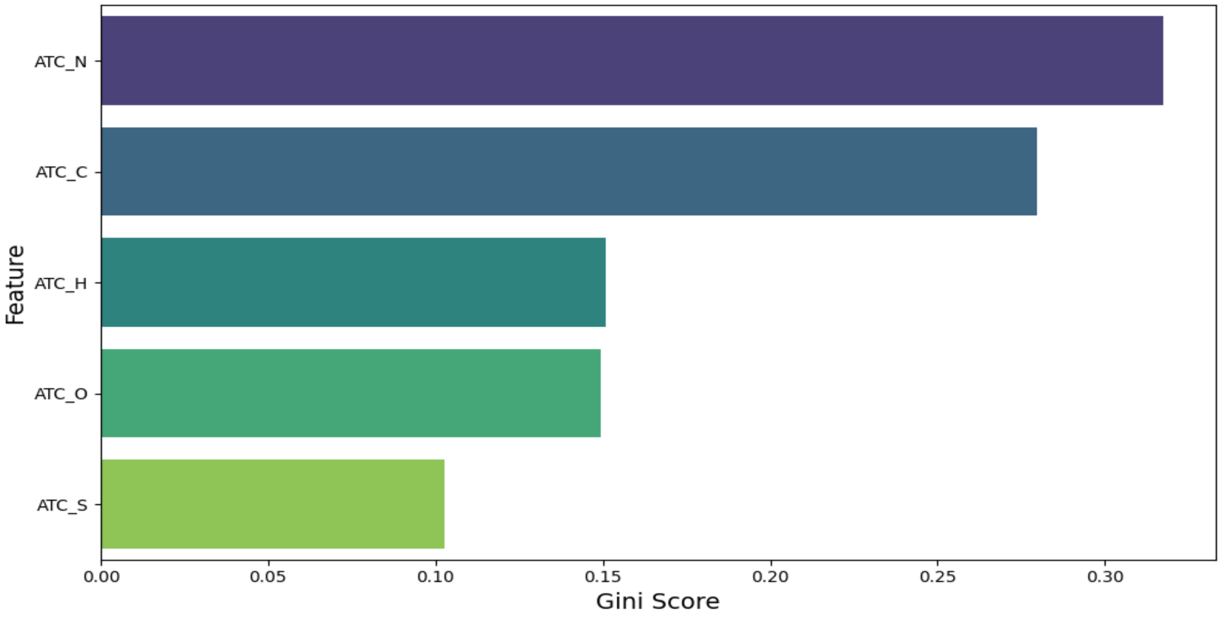

Figure S2: Feature Importance of Atom Type Composition

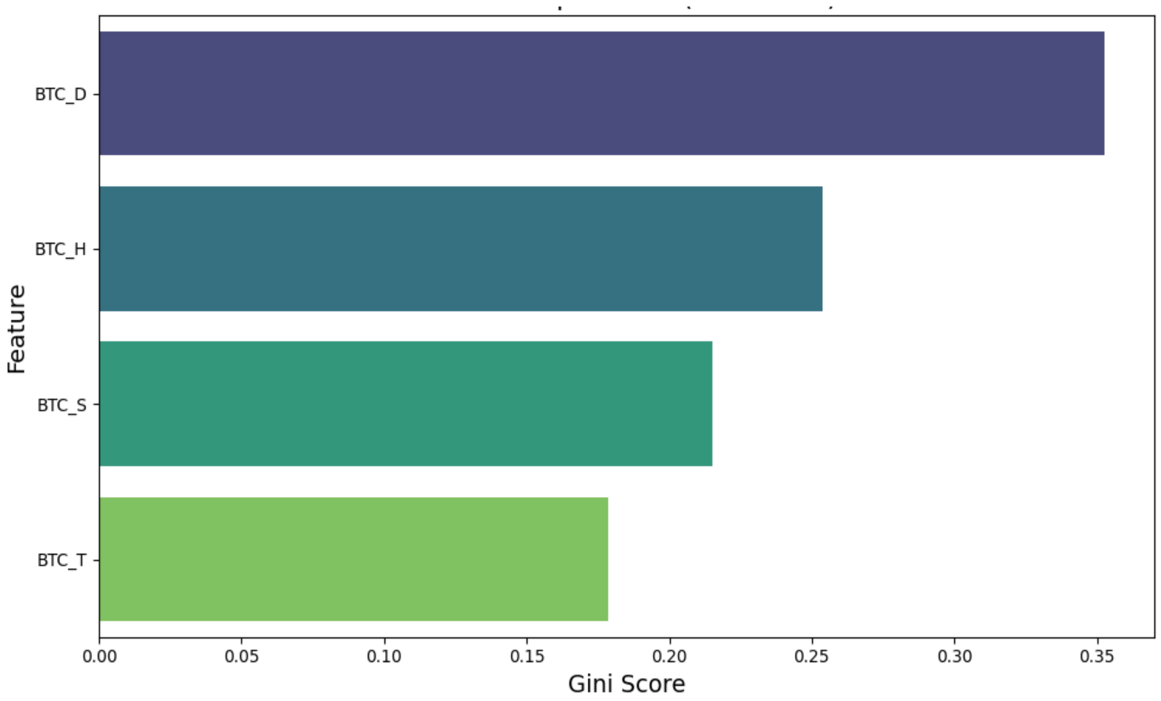

Figure S3: Gini Score of Bond Type Composition

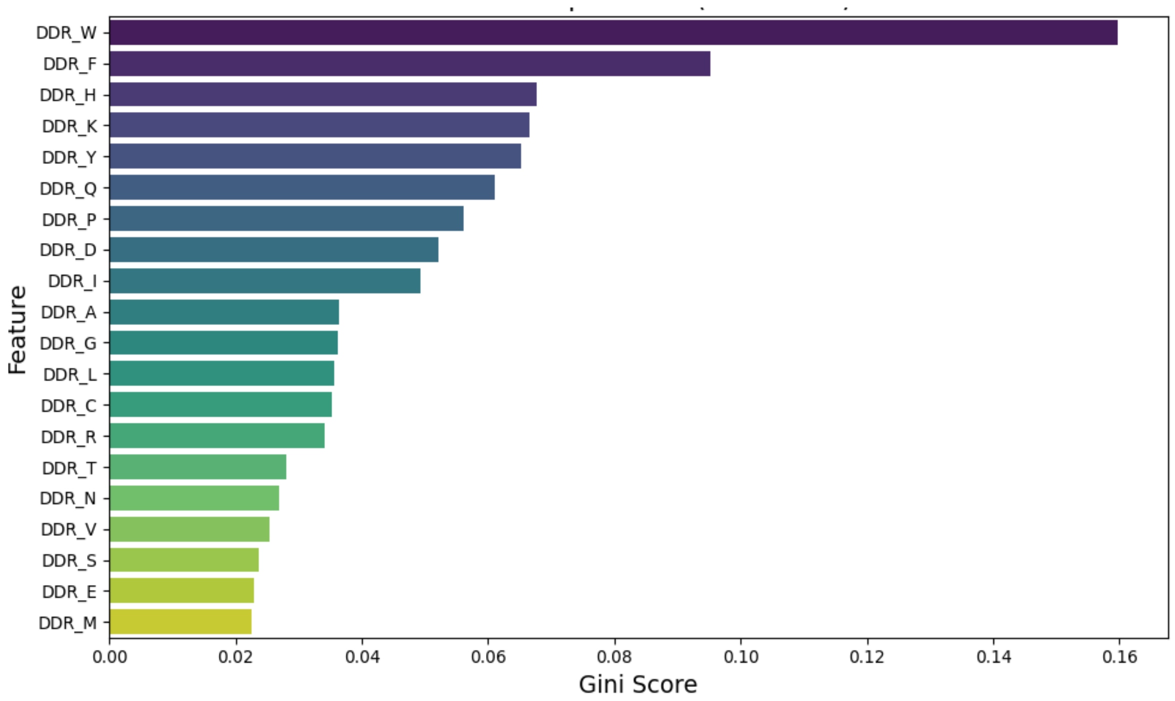

Figure S4: Gini Score of Distance Distribution of Residues

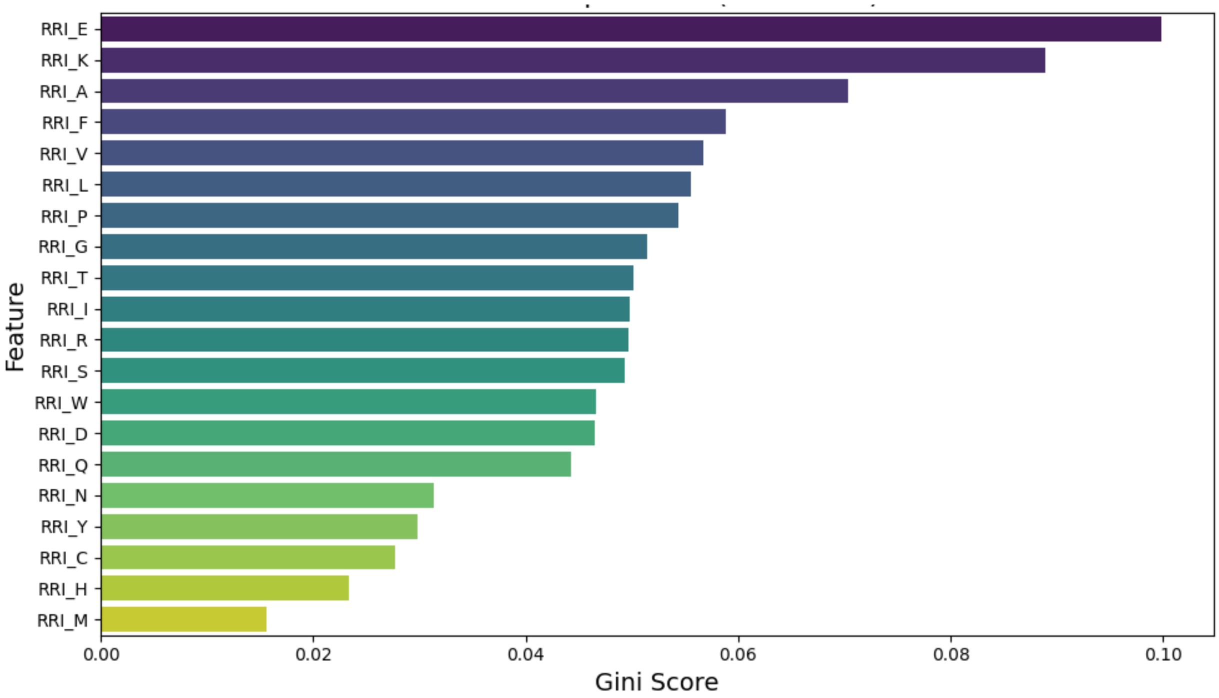

Figure S5: Gini Score of Repeat Residue Information

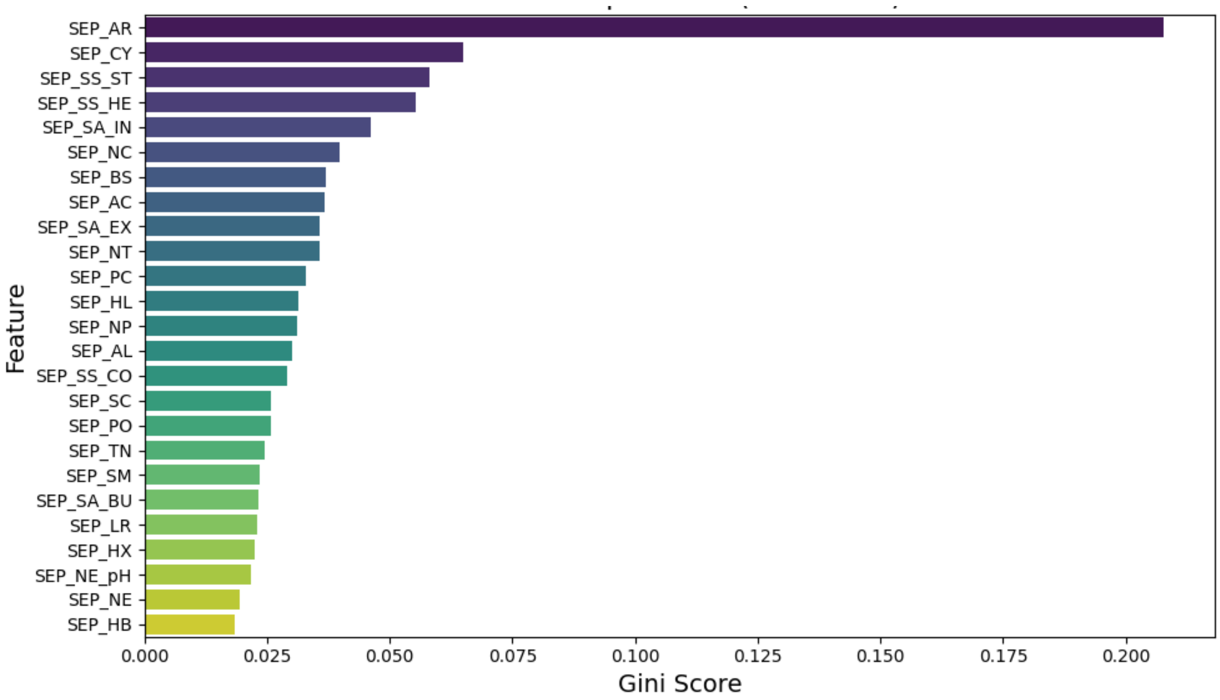

Figure S6: Gini Score of Shannon Entropy of Physicochemical Properties

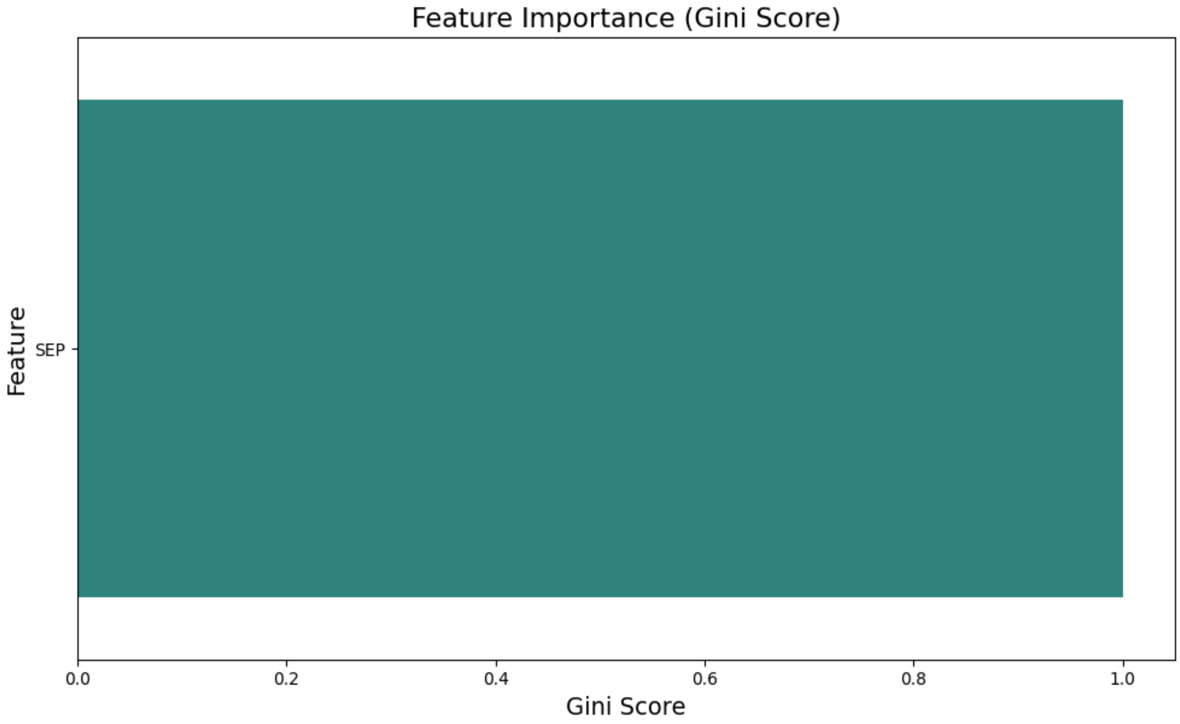

Figure S7: Gini Score of Shannon Entropy
